# Two Growing-Season Warming Partly Promoted Growth but Decreased Reproduction and Ornamental Value of *Impatiens oxyanthera*

**DOI:** 10.3390/plants13040511

**Published:** 2024-02-12

**Authors:** Jiayu Tao, Youqin Yang, Qiong Wang

**Affiliations:** Southwest Key Laboratory of Wildlife Conservation (Ministry of Education), China West Normal University, Nanchong 637009, China; t874761126@163.com (J.T.); 18387884183@163.com (Y.Y.)

**Keywords:** climate warming, *Impatiens* L., plant morphology, floral traits, ornamental value, analytic hierarchy process

## Abstract

Climate warming profoundly affects the vegetative growth, flowering phenology and sexual reproduction of plants; therefore, it affects the ornamental value of wild flowers. Despite this, the extent and mechanism of the impact remain unclear. Here, we conducted a warming experiment for two growing seasons (increases of 1.89 °C in 2017 and 2.37 °C in 2018) with infrared heaters to examine the effects of warming on the ornamental value of the wild flower *Impatiens oxyanthera*, endemic to China, in Mount Emei. We evaluated the comprehensive ornamental value based on plant morphology and flowering characteristics using the analytic hierarchy process (AHP) and disentangled the impact of the two traits on ornamental value using principal component analysis (PCA) and the partial least squares structural equation model (PLS-SEM) under ambient and warming treatments. We hypothesized that warming would reduce the ornamental value of *I. oxyanthera* in terms of plant morphology and flowering traits. Our results showed that warming significantly decreased plant height and crown width and increased branch number and single-leaf area. Warming also decreased vexillum length, corolla tube length, nectar spur length and pedicel length. In addition, warming shortened flowering duration per plant and reduced flower number, while there was no significant effect on flower longevity and flower color at full-bloom stage between the control and warming treatment. Therefore, the comprehensive ornamental value under warming was lower than that under the control. Pedicel length, flower color, flower longevity and flowering duration per plant were the main factors affecting the comprehensive ornamental value. The PLS-SEM showed that warming had an indirect negative effect on ornamental value via direct negative effects on flowering traits. Collectively, these results indicate that, although promoting vegetative growth, short-term warming significantly decreased the ornamental value of *I. oxyanthera* due to warming-caused smaller flowers and shorter flowering duration.

## 1. Introduction

Flowering plants are the most highly diverse plant group with approximately 350,000 species. These plants have notably shaped terrestrial landscapes because they make up 90% of all living land plant species and their flowers color and scent the world [1]. These flowering plants are sensitive to the increase in temperatures. Nowadays, the mean global surface temperature have increased by 1.25 °C since 1850 to 1900 and will exceed 1.5 °C in less than 10 years, according to the current emissions trajectory of greenhouse gases [2]. Exceeding 1.5 °C global warming could trigger multiple climate tipping points [3]. Temperature, as the survival condition, affects the growth, morphology and reproduction of plants [4,5,6,7], thereby altering species survival and population maintenance and ultimately affecting community biodiversity. Although intrinsic climate adaptations may buffer species from local extinction to varying degrees [8,9], a large body of research suggests that many species are at risk of becoming extinct as a result of climate change [10,11,12] and that this risk increases with warming [13,14,15]. It can be predicted that climate warming will threaten the survival and reproduction of plants, including wild flowers [16], and thus determine their ornamental value because weather or climate factors (air temperature, precipitation) affect the ornamental traits of plants [17,18]. Therefore, studies on the effects of warming on the growth and reproduction of wild flowers can help to predict population dynamics and distribution and provide a theoretical basis for the development of rational species conservation strategies under future climate change.

Plant type and leaf shape at the vegetative growth stage are important indicators to evaluate a plant’s ornamental value. Many plants respond to a temperature increase by altering their activity and metabolism [19]. Warming generally has a positive effect on plant growth due to increased photosynthesis and the accumulation of dry matters when ambient temperature is lower than the optimal temperature of the plant [4,20]. In addition, warming can affect the relative content of plant hormones [21], which then influences plant morphology and growth. A higher than optimal temperature inhibits the apical dominance of plants and promotes plant branching because of the vigorous activities of the lateral meristem. Besides the traits of vegetative growth, reproductive traits are core indicators to evaluate the ornamental value of flowering plants [22]. Temperature profoundly affects plant flowering by directly influencing flower induction and development and indirectly affecting resource allocation between vegetative growth and sexual reproduction [23]. Climate warming leads to a significant reduction in flower density at the landscape level and flower number or flowering likelihood at the individual level [24,25] because elevated temperatures cannot meet the low-temperature requirements for vernalization of flowering species or cause serious flower bud abortion. However, it was found that the model plant *Arabidopsis thaliana* responded to a warming environment by accelerating vegetative growth and increasing flower number [26]. Moreover, warmed plants produced smaller flowers as a result of the limitation of higher temperature on flower development [27,28]. At the individual level, temperature affects anthocyanin synthesis, color reaction and anthocyanin stability [29]. For flowering plants, the flower longevity and flowering duration of individuals or groups determine the length of the ornamental period and plant reproductive success [30]. Temperature affects flower longevity by changing the cost of keeping flowers open [31]. High temperature accelerates the respiration rate of flowers and water evapotranspiration, leading to an accelerated senescence of the flowers [32], thus shortening flower longevity. Altogether, the effects of warming on plant growth and sexual reproduction are often brought forward, but few comprehensive evaluations of the ornamental value for wild flowers exist in responses to warming.

*Impatiens* L. have a higher ornamental value due to their diverse colors, unique flower shape and long flowering period. The genus has more than 900 known species and is widely distributed all over the world [33]. There are approximately 220 species known in China, and it is one of the famous traditional flowers in China and even the world [34]. Wild plants of this genus can provide excellent germplasm resources for garden flowers and have great development potential. However, only *Impatiens wallerana* Hook. f. and *Impatiens hawkeri* W. Bull were cultivated worldwide [35]. The ornamental characters and utilization value of 40 wild *Impatiens* species were evaluated comprehensively by using the analytic hierarchy process [36]; warming delayed lowering onset, shortened flowering duration and reduced flower size of some wild *Impatiens* species [37]. Because *Impatiens* spp. are one of the important components of understory vegetation [28], the hypothesis of this study is that warming will reduce the ornamental value of *Impatiens* spp. by affecting its plant morphology and floral characteristics. Therefore, *Impatiens oxyanthera* Hook. f., a perennial herb endemic to China, was taken as the study plant.

## 2. Results

### 2.1. Plant Vegetative Growth

**Figure 1:**
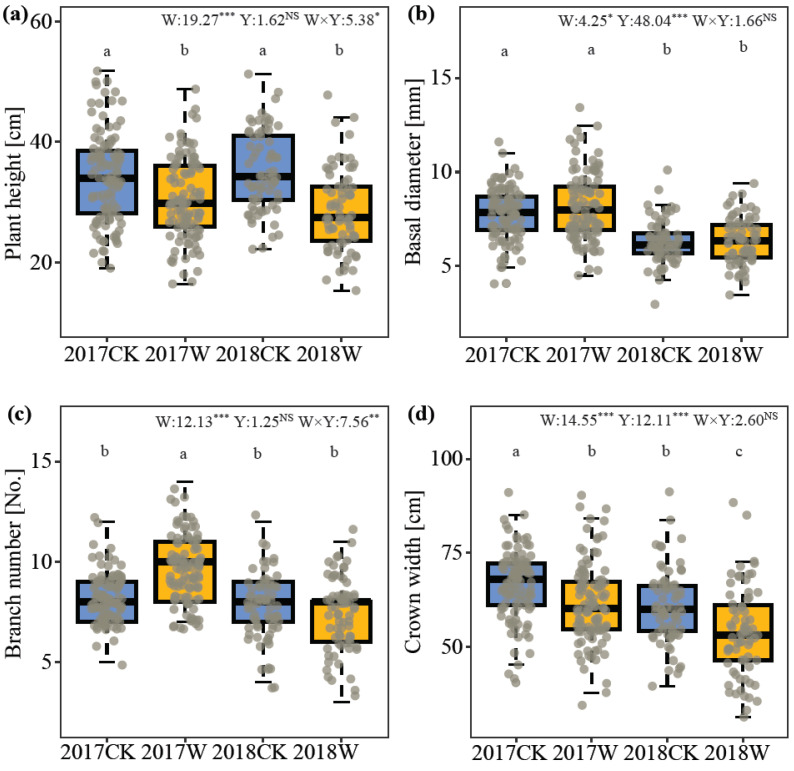

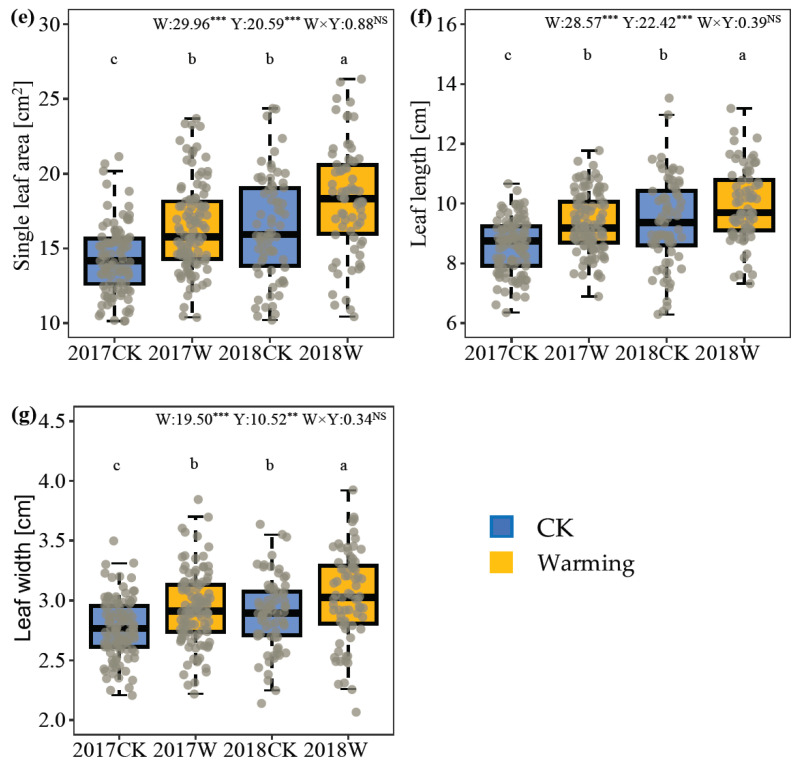
Effects of simulated warming (W: control and warming) and year (Y: 2017 and 2018) on plant morphology of *I. oxyanthera*. (**a**)**.** Plant height, (**b**)**.** Basal diameter, (**c**)**.** Branch number, (**d**)**.** Crown width, (**e**)**.** Single leaf area. (**f**)**.** Leaf length. (**g**)**.** Leaf width. W, the effect of warming; Y, the effect of year; W × Y, the interaction effect of warming and year. Different lowercases represent a significant difference among the four experimental treatments. NS, no significance; *, *p* < 0.05; **, *p* < 0.01; ***, *p* < 0.001.

Warming significantly changed the vegetative growth of *I. oxyanthera* in 2017 and 2018. Plant height and crown breadth were considerably restricted by warming. Plant height under warming was decreased by 12.04% in 2017 and by 18.85% in 2018 (Figure 1a). The plants had a smaller crown width (reduction of 7.43% in 2017 and 11.76% in 2018) under warming (Figure 1d). In contrast, branch number, leaf area, leaf length and leaf width increased under warming. For branch number, warming significantly promoted branching in *I. oxyanthera* only in 2017, increasing by 17.27% (Figure 1c). Warming improved leaf area by 15.62% in 2017 and by 11.17% in 2018 (Figure 1e). Warmed plants had longer (8.48% and 6.07% increases in 2017 and 2018, respectively) and wider (5.88% and 4.67% increases in 2017 and 2018, respectively) leaves (Figure 1f,g). Similarly, the year also significantly affected the vegetative growth of *I. oxyanthera*, except plant height and branch number. Compared with 2017, basal diameter and crown width were significantly reduced by 20.60% and 10.63% in 2018, respectively (Figure 1b,d). However, single-leaf area in 2018 increased significantly by 11.96% (Figure 1e). And plants in 2018 had longer (7.80% increase) and wider (3.89% increase) leaves than those in 2017 (Figure 1f,g). Their interaction had a significant effect on plant height and branch number. In 2017, warming significantly increased the number of branches, while there was no effect in 2018.

### 2.2. Flower Longevity and Flowering Duration

The year had a significant effect on the male phase and flower longevity but not the female phase. The male phase of flowers and single-flower longevity in 2018 were significantly higher (19.12% and 21.68%, respectively) than those in 2017. However, warming and the interaction of warming and year had no effect on flower longevity. At the individual level, warming significantly shortened flowering duration per plant, shortening by 9.70% in 2017 and by 19.05% in 2018. Warming had a significant effect on flower number per plant in the full-flowering period of *I. oxyanthera*; warmed plants had fewer flowers than the control plants in 2017 (9.25% reduction) and 2018 (10.85% reduction). Similarly, the year had a significant effect on the flower number per plant in the full-flowering period of *I. oxyanthera*, and the flower number per plant in 2018 was 9.81%, which was less than that in 2017 (Table 1).

### 2.3. Floral Traits

Two growing-season warming had different effects on floral traits. Simulated warming caused a smaller flower and shorter pedicel. Warming significantly reduced the vexillum length, wing petal length, corolla tube length, nectar spur length and pedicel length, decreasing by 7.33%, 3.34%, 4.59%, 8.75% and 16.47% in 2017, respectively. In 2018, compared with the control group, the wing petal length and corolla tube length decreased by 3.56% and 8.84%, respectively. Some traits of flower morphology significantly changed with the year. The corolla diameter and nectar spur curvature in 2018 were higher than those in 2017, increasing by 5.41% and 9.52%, respectively. In contrast, the flowers in 2018 had a shorter nectar spur length and pedicel length compared to the flowers in 2017, decreasing by 2.16% and 6.99%, respectively. The interaction of warming and year had a significant effect on the nectar spur length and pedicel length. Warming in 2017 and 2018 had no significant effect on the relative anthocyanin content of the vexillum and corolla tube (Table 2).

### 2.4. Comprehensive Ornamental Value

**Figure 2:**
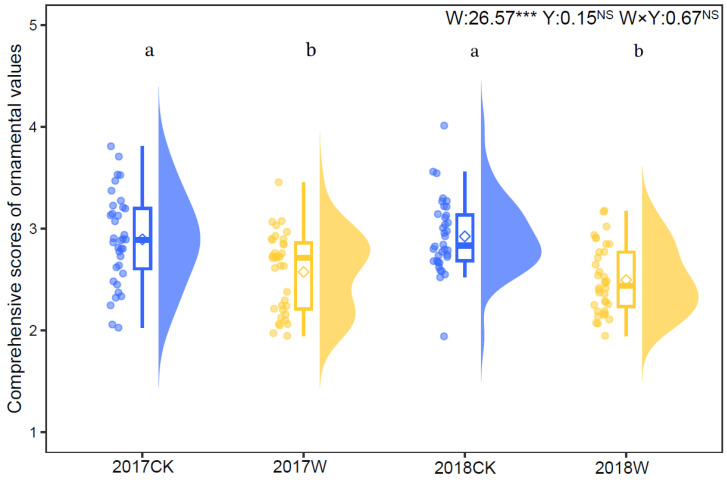
Raw data, boxplots and density of data points for comprehensive scores of ornamental values of *I. oxyanthera* under the control and warming treatment in 2017 and 2018. Diamonds indicate mean values. W, the effect of warming; Y, the effect of year; W × Y, the interaction effect of warming and year. Different lowercases represent a significant difference among the four experimental treatments. NS, no significance; ***, *p* < 0.001.

The analytic hierarchy process (AHP) showed that the comprehensive score of the ornamental values was lower under warming (2.575 ± 0.064) compared with the control (2.896 ± 0.074) in 2017. In 2018, the comprehensive score of the ornamental values under warming (2.496 ± 0.055) was also lower than that under the control (2.925 ± 0.061). Warming significantly decreased the comprehensive score of *I. oxyanthera* by 11.08% in 2017 (*t* = 3.878, *p* < 0.05) and by 4.67% in 2018 (*t* = 5.083, *p* < 0.05). The year and the interaction of warming and year had no significant impact on ornamental value (Figure 2).

**Figure 3:**
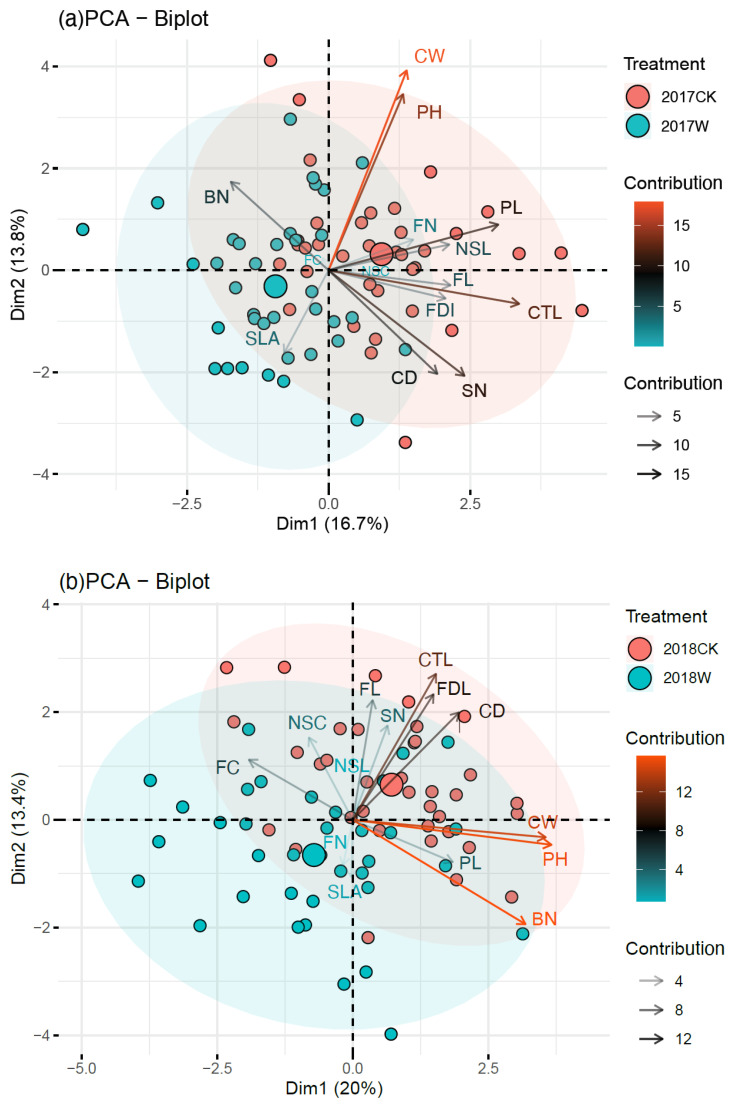
Principal component analysis (PCA) of ornamental indicators in 2017 (**a**) and in 2018 (**b**)**.** PH, plant height; BN, branch number; CW, crown width; SLA, single-leaf area; FL, flower longevity; FC, floral color; FDI, flowering duration of individual; FN, flower number; CTL, corolla tube length; NSL, nectar spur length; NSC, nectar spur curvature; SN, stripe number on the labellum; PL, pedicel length; CD, corolla diameter.

The results of the PCA showed that the variance interpretation rates of principal components 1 and 2 accounted for 16.70% and 13.80% of the total variance of all traits, respectively, with a total of 30.50% in 2017 (Figure 3a). The variance interpretation rates of principal components 1 and 2 were 20.00% and 13.40% of the total variance of all traits, respectively, with a total of 33.40% in 2018 (Figure 3b). In 2017, the first principal component was mainly composed of the corolla tube length (CTL), pedicel length (PL), stripe number on the labellum (SN), branch number (BN) and single-leaf area (SLA), which was positively correlated with CTL, PL and SN and negatively correlated with BN and SLA. The second principal component was highly correlated with the crown width (CW), plant height (PH), stripe number (SN), corolla diameter (CD) and other traits, which was positively correlated with CW and PH and negatively correlated with SN and CD (Figure 3a). In 2018, the first principal component was mainly correlated with the PH, CW, BN, floral color (FC), nectar spur curvature (NSC) and other traits, which was positively correlated with PH, CW, and BN and negatively correlated with FC and NSC. The second principal component was mainly composed of the CTL, FH, FL, BN, SLA and other traits. Among them, it was positively correlated with CTL, FH and FL and negatively correlated with BN and SLA (Figure 3b).

**Figure 4:**
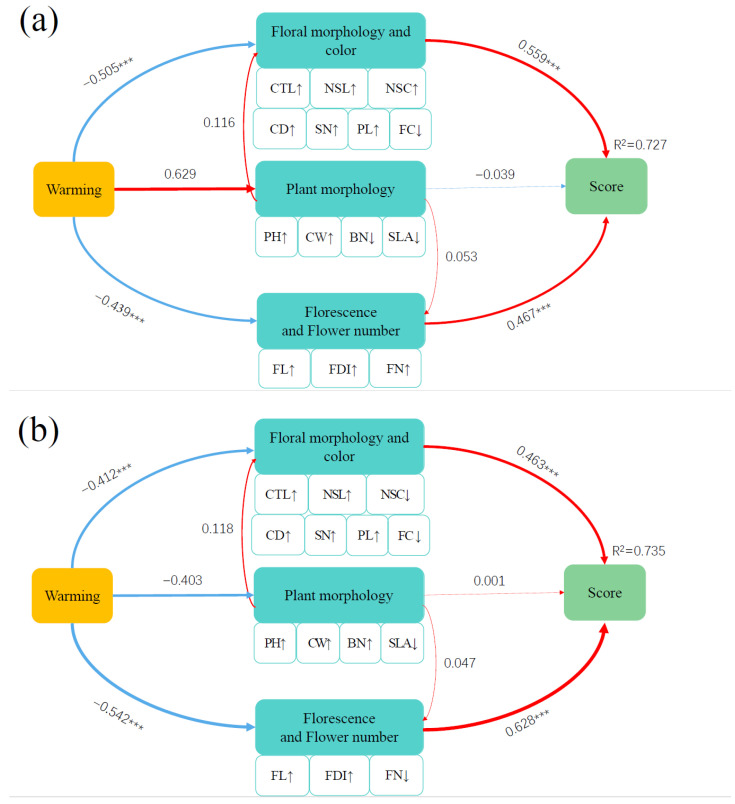
Partial least squares structural equation model (PLS-SEM) in 2017 (**a**) and in 2018 (**b**)**.** Partial least squares structural equation model (PLS-SEM) depicting the effects of warming on comprehensive score of ornamental value of *I. oxyanthera* through direct effects on plant morphology, floral morphology and color and florescence and flower number. Single-headed arrows indicate the direction of a hypothetical causal relationship. Red and blue arrows indicate positive and negative relationships, respectively. Arrow width is proportional to the strength of the correlation. Double-layer rectangles represent the first component of PCA. The symbols ‘↑’ and ‘↓’ represent the positive and negative correlations between variables and the first component of PCA, respectively. *R*^2^ is the proportion of variance. The number next to the arrow is the standardized path coefficient. Significant path coefficients are marked with asterisks: ***, *p* < 0.001. No asterisks means no significance.

The PLS-SEM integrated the direct and indirect effects of the studied plant morphology and flowering variables on the comprehensive ornamental value under warming, explaining 30.50% and 33.40% of the variation in the effects of plant morphology and flowering traits on ornamental value under warming in 2017 and 2018, respectively (Figure 4). The results of the PLS-SEM in 2017 showed that warming had an indirect effect on the comprehensive score of the ornamental value through direct positive effects on plant morphology and direct negative effects on flowering traits (Figure 4a). The results of the PLS-SEM in 2018 showed that warming had an indirect effect on the comprehensive score of the ornamental value through direct negative effects on plant morphology and flowering traits (Figure 4b).

## 3. Discussion

Our results demonstrate that warming can dwarf plants, promote branching and enlarge leaf area of *I. oxyanthera* at vegetative growth stages but shorten flowering duration at the flower and individual level and decrease flower size and flower stripe number. Thus, our results indicate that short-term stimulated warming had a negative effect on the comprehensive ornamental value of *I. oxyanthera*. These results imply that climate warming will decrease the ornamental value of wild herbaceous flowers in the short-term.

### 3.1. Effect of Warming on Plant Vegetative Growth

The apical meristem is responsible for main stem growth (plant height), whereas the axillary meristem is responsible for lateral branching (branch number). In this study, two-year warming significantly decreased plant height and crown width but increased the number of primary branches in 2017. Warming inhibited the growth of the stem in *I. oxyanthera*, consistent with the responses of the invasive plant Solidago canadensis to warming [38]. On the one hand, apical dominance is temperature-dependent [39]. On the other hand, heat stress is usually accompanied by a water deficit. Both heat and water stress influence the activities of PSII and PSI and, thereby, plant growth and viability [40]. In this study, warming in 2017 promoted a number of primary branches of *I. oxyanthera*, which may indicate that moderate warming increased the relative content of cytokinins and promoted axillary bud growth [41]. However, warming in 2018 had no significant effect on the number of primary branches, probably because the plants had adapted to the warmed environments in 2018 after the first year of warming. Warming significantly increased the single-leaf length, width and area of *I. oxyanthera*. Studies have shown that warming in the normal season increases leaf biomass allocation and thus promotes the growth of leaves [42], which help plants effectively capture light energy and maintain photosynthesis [43]. Warming-caused lower stems and increased branches make the plants low and dense, which will increase the difficulty of foraging for flowers and reduce the foraging efficiency of pollinators [44]. However, warming-caused dwarfing has many advantages in landscape application because of high space utilization, low pruning frequency and lodging resistance. The increase in branch number and leaf area is beneficial to the formation of a larger photosynthetic area [45], but the increase in leaf area under warming can improve the transpiration water loss, which then aggravates the water deficit of plants.

### 3.2. Effect of Warming on the Ornamental Time and Flowering Period

In our study, warming had a significant negative effect on flowering duration at the single flower and individual level in *I. oxyanthera*. Warming shortened the male phase of the flower but not the female phase of the flower, thus shortening single-flower longevity. Flower longevity is easily affected by temperature [31]. The optimum temperature range for the development of male organs is narrower than that of female organs [46], which may be the reason why the male phase is more sensitive to increased temperatures than the female phase of *I. oxyanthera* in this study. The shortening of the male phase may reduce pollen dispersal and male reproductive success [47]. It has been found that high temperature shortens flower longevity because of a faster respiration rate and the higher energy cost for maintaining flowers [32,48]. The delay in first-flowering time together with a shortening of flowering duration in *I. oxyanthera* suggest a negative impact on pollinators, which might pose a threat to plant reproductive success [49]. Meanwhile, the shortened flowering duration of an individual plant can cause a reduction in the ornamental value of plants.

### 3.3. Effect of Warming on Flower Ornamental Characteristics

Warming had a significant negative effect on the floral traits of *I. oxyanthera*, except flower color. The flowers of *I. oxyanthera* under warming had a shorter corolla tube, vexilla and wing petal, consistent with the warming-driven smaller flower in the previous study [27]. Several factors may have contributed to this result. First, the higher temperature directly inhibited the development of flowers due to reduced cell division [50]. Second, the reduction in flower size under heat stress could result from decreased photosynthesis and assimilate supply to the flowers [51]. Third, when plants are under heat stress, reproductive investment will reduce as available resources decline and the probability of mortality increases [52]. Hence, decreased flower size rather than flower number occurred in *I. oxyanthera.* Finally, increased temperatures reduce atmospheric humidity (Figure S1), promote flower transpiration and water loss and require more water to maintain the flower display [53]. Therefore, the small corolla under warming can reduce the reproductive costs of plants. However, the smaller corolla is not easily selected by pollinators [54], thereby limiting pollination success. A long pedicel facilitates the flower display and pollinator’s visiting, but warming significantly shortened the pedicel length of *I. oxyanthera*, especially in 2017. A long and curved nectar spur is not only beautiful in shape but also can increase the contact between pollinators and flowers, thereby improving plant reproductive success [55,56]. Warming shortened the nectar spur of *I. oxyanthera*, especially in 2017, and had no effect on nectar spur curvature, which might reduce the difficulty of insects sucking nectar and the tightness of long-mouthed pollinators in contact with anthers or stigmas, thereby reducing pollination success [57]. The stripe number on the labellum of *I. oxyanthera* can increase its ornamental value but was decreased under warming. Bright colors have strong attraction, but warming has no significant effect on the anthocyanin content of the flowers in *I. oxyanthera*. The optimal temperature for anthocyanin accumulation varies with species. In this study, the warming magnitude was 1.9–2.4 °C, which may not exceed the optimal temperature for anthocyanin biosynthesis in *I. oxyanthera*.

### 3.4. Effect of Warming on Comprehensive Ornamental Value

The analytic hierarchy process (AHP) simplifies complex problems by using hierarchical methods, which not only contain subjective logical judgment but also make full use of the advantages of quantitative analysis [58,59]. It plays an important role in screening plant resource varieties [60,61,62] and evaluating ornamental value [36]. In previous studies, the ornamental value of multiple species was evaluated by using AHP [63]. Our study first applied AHP to evaluate and compare the comprehensive ornamental value of a flowering plant between different warming conditions. And it was concluded that flowering traits were the most important limiting factor of ornamental value, which was consistent with Wang’s result [36]. Plant morphology parameters were the smallest limiting factor (Figure 3). Among the 15 selected evaluation factors, pedicel length, floral color, individual flower longevity and flowering duration per plant have the greatest effect on the ornamental value of *I. oxyanthera*. Most of these indexes under warming were significantly lower than those under the control, thus leading to the decrease in the ornamental value of *I. oxyanthera*.

PCA was used to screen-out the indexes with high ornamental value, including floral color, flowering duration per plant, individual flower longevity, pedicel length, leaf area and so on. We used the outcomes of PCA for the PLS-SEM to determine the relationship between the vegetative growth, reproductive growth, florescence and comprehensive score of ornamental value. The PLS-SEM provides strong evidence that warming had an indirect negative effect on ornamental value via direct negative effects on flowering traits. Ye [64] found that flower morphology is the core factor in evaluating the ornamental value of *C.ensifolium* cultivars, which is consistent with the results of our study. Warming significantly reduced the flower size of *I. oxyanthera* so that the ornamental value of *I. oxyanthera* decreased. Moreover, warming significantly reduced flower number and shortened the florescence, which greatly reduced the ornamental value and ornamental cycle [65].

## 4. Materials and Methods

### 4.1. Study Site and Plant Materials

The experimental site is located on Mount Emei in China (29°36.16 N, 103°21.62′ E, a.s.l. 932 m), a transition zone between the southwest edge of the Sichuan Basin and the Qinghai-Tibet Plateau and a climate sensitive area [66]. It is a subtropical, monsoon, humid climate with four distinct seasons. The average annual temperature is 10–17 °C, and the average annual rainfall is 1593–1990 mm. The soil in the region is yellow soil [67]. The experimental site is mainly located in the evergreen broad-leaved forest belt [68], and the dominant plants belong to Lauraceae and Fagaceae. Emei Mountain is one of the important distribution and differentiation regions of the *Impatiens* species in China. There are 24 species of wild *Impatiens*, 9 of which are endemic species, mostly distributed at the altitude of 500–3000 m [69].

*I. oxyanthera* is a perennial herb endemic to China, distributed on forest edges and roadsides between 800 and 3000 m above sea level on Mount Emei [69,70]. Flowering occurs in late summer and autumn from August to October. The flowers are big and red or reddish-lavender. The labellum of the flower is funnel-shaped with some red stripes. The base of the labellum has a long and curved nectar spur [33]. Thus, *I. oxyanthera* has a higher ornamental value [36].

### 4.2. Warming Treatment

In March 2017, 432 wild seedlings of *I. oxyanthera* with an approximately 10 cm height were transplanted from nearby natural habitats into 10 L plastic pots filled with local soil. They were randomly assigned to twelve 2 m × 2 m experimental plots (6 rows and 6 columns, a total of 36 seedlings per plot). The interval between the experimental plots was 1 m. The twelve plots were randomly assigned to two experimental treatments (increased temperatures and the control) with 6 plots in each treatment. The warming was achieved by hanging 165 cm × 15 cm infrared heaters (Kalglo Electronics Inc., Kalglo, PA, USA) with a power of 2000 W at a height of 2 m above the ground. In order to simulate the shading effect of the heater, a wood board with the same projected area as the infrared heater was hung directly above the control plot. Meanwhile, the infrared heater (or wood board) was rotated 45° clockwise every 10 days. In order to simulate the relative light intensity of the native habitat of *I. oxyanthera*, a layer of black sunshade net was covered above the experimental plot with a transmittance of (26.83 ± 0.66) % at a height of 3 m above the ground. All-day warming was carried out, lasting from April 22 to late October 26, 2017. The average daily air temperature under the warming conditions (22.86 ± 0.32 °C) was increased by 1.89 °C above ambient temperatures (20.97 ± 0.29 °C) (Figure S1a, Table S3). At the end of March 2018, only one healthy branch from the old stem with similar growth status was kept, and the other branches were removed in each flowerpot. During the second growing season, from 9 April to 25 October 2018, the average daily air temperature under the warming conditions (22.68 ± 0.34 °C) was increased by 2.37 °C above ambient temperatures (20.31 ± 0.29 °C) (Figure S1b, Table S3). The magnitude of warming was set based on the predicted increase in average global temperatures in the IPCC report [71]. The irrigation pattern was rain-fed and manual irrigation with manual watering in the morning when the soil became dry during many consecutive sunny days.

### 4.3. Determination of Air Temperature, Humidity and Soil Temperature

A temperature and humidity recorder (DS1923G, Maxim/Dallas Semiconductor Inc., Wilmington, MA, USA) was installed at the middle of the second or fourth rows in each plot. It was the same height as the plant and placed symmetrically in every two plots to measure air temperature and relative humidity. Temperature sensors (DS1921G-F5, Maxim/Dallas Semiconductor Inc., Wilmington, MA, USA) were used to monitor the soil temperature. Because *I. oxyanthera* is a shallow-root plant, the temperature sensor was placed in the first flowerpot on the right of the temperature and humidity recorder under the soil at a depth of 10 cm. The data of temperatures and relative humidity were automatically logged every hour throughout the six-month warming experiment for each year.

### 4.4. Measurement of Plant Morphology

In July 2017 and 2018 before the plants bloomed, 108 plants were randomly selected in the control and warming treatments, respectively. Plant height was the vertical distance from the base to the top of the stem, and the basal diameter of the stem was the diameter of the stem near the soil with a digital vernier caliper (Japan Sanfeng Mitutoyo 500-153, accuracy 0.01 mm, Shenzhen Baoan Tengyueda Electronic Tools Co., Ltd. Shenzhen, CN). Crown width was represented by the average length of lateral branch coverage in two fixed directions perpendicular to each other on the sample plant. Branch number was counted for branches longer than 10 cm. At the same time, five mature leaves were randomly selected from each plant in the same direction. Leaf area was measured with a leaf area analyzer (Top YMJ-C, Zhejiang Top Instrument Co., Ltd. Hangzhou, CN), and the length and width of the leaves were measured.

### 4.5. Determination of Ornamental Traits of the Flower

From August to October in 2017 and 2018, 36 plants were randomly selected from the plants whose plant morphology had been measured under the two warming treatments, respectively. And the data of first and final flowering for the target plants were recorded; then, the flowering duration of the individual plants was calculated. The number of flowers was counted during the full-flowering stage. Meanwhile, three mature flower buds were randomly selected from the middle and upper part of these plants to observe the duration time of the male and female phases and flower longevity. Because the flowers of *I. oxyanthera* keep the same size before withering, three male-phase flowers of the object plants were randomly selected to measure floral traits, including corolla tube length, nectar spur length, number of stripes on the labellum, nectar spur curvature, vexillum length, wing petal length and corolla diameter. The measurement standard is shown in Figure S2.

### 4.6. Determination of Anthocyanin Content

Relative anthocyanin content was measured with a modified methanol hydrochloride spectrophotometer in October, 2017 and 2018. Flowers were collected from 72 plants in which floral characteristics had been measured. We washed the fresh flowers with distilled water, and then drained the distilled water with a filter paper, cut the vexillum and corolla tube (including the wing petal and labellum) into pieces, weighed 0.100 g of petal and added them to a vial containing 9 mL 1% methanol hydrochloride. We measured the absorbance of the solution with a UV–visible spectrophotometer at the wavelength of 530 nm after the petal was soaked for 48 h. The relative anthocyanin content was divided by the absorbance of fresh weight 0.100 g [72].

### 4.7. Evaluation of Ornamental Value

The ornamental value of the above-mentioned 36 plants in each treatment per year under the control and warming treatments in 2017 and 2018 was evaluated with reference to Wang’s method [36].

**Figure 5:**
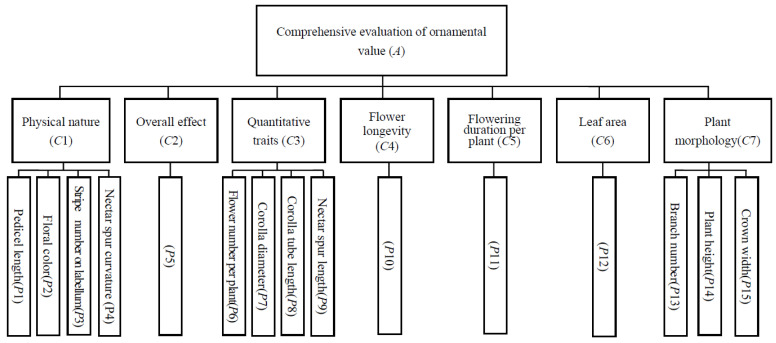
Hierarchy evaluation model for evaluation of comprehensive ornamental value of *oxyanthera* under the control and warming.

First, a comprehensive evaluation model was established based on plant morphology and flower ornamental characteristics. The evaluation model was divided into three hierarchies. The first hierarchy was target hierarchy A, which was the comprehensive score obtained after evaluating different indexes of all target plants. The second hierarchy was constraint hierarchy C, which was the main ornamental traits involved in the evaluation, including physical properties, overall effect, quantitative traits, floral longevity, flowering duration per plant and leaf and plant morphology. The third hierarchy was standard hierarchy P, which was 15 specific evaluation indexes of each character belonging to hierarchy C (Figure 5).

Second, the judgement of matrix construction and the check of consistency were conducted. The relative importance of corresponding factors in two adjacent layers was quantified by the ratio scale method of 1, 3, 5, 7 and 9, and a judgment matrix was formed. The matrix consistency ratio was calculated by using the formula *CR* = *CI*/*RI*, where *CR* represents the random consistency ratio, *CI* is the indicator of deviation from consistency of the judgment matrix (*CI* = (*λ_max_* − *n*)/(*n* − 1), *λ_max_* is the maximum eigenvalue of the judgment matrix, and *n* is the order of the judgment matrix), and *RI* is the average random consistency indicator of the judgment matrix (Table S1). If *CR* < 0.100, the judgment matrix is considered to have satisfactory consistency; otherwise, it should be adjusted. The four matrices were tested with satisfactory consistency (*CR* < 0.100, Table S2).

Finally, a total hierarchical sort calculation was performed. The total ranking weight value of each evaluation index in the standard hierarchy P relative to the target hierarchy A is the weighted value of each index in the standard hierarchy P relative to the corresponding constraint hierarchy C, and the weight of constraint hierarchy C is weighted and integrated (Table S2).

### 4.8. Data Analysis

Statistical analyses were conducted with R version 4.3.0 (R Core Team, 2021). The residuals of air temperature, air humidity and soil temperature were not normally distributed. Accordingly, we assessed the two growing-season warming using GLMMs (Gamma distribution with a log link), and the random effect was date ID (day of year). To examine how the plant morphology and floral traits differed between the warming and the control, we fitted generalized linear mixed models (GLMMs) (Gamma or Poisson distribution with a log link) using the fixed effects of treatments (i.e., warming and year) and the random effect of plant ID nested in plot ID. We performed a generalized linear model (GLM) with Poisson and log-link function to determine the effects of warming and year on flower longevity (include male phase and female phase). To assess the effects of the warming treatment on the comprehensive scores of ornamental values, we fitted GLMM (Gamma distribution) using plant ID as a random effect for the data of 2017 and fitted the linear regression model for the data of 2018. The GLMMs and GLM were performed using the R package of *lme4* [73] and statistical data, respectively. The type III Wald χ^2^ ANOVA test was used in the R package of *car* to determine the statistical significance of the effect [74]. In order to compare the different treatment combinations in the analysis, the contrast of the estimated marginal mean (adjustment method: Tukey) was calculated in the R package of *emmeans* [75]. When the ANOVA was not significant, the Tukey test was not performed.

We used the *FactoMineR* package to perform principal component analysis (PCA) on the indicators with higher weights and more comprehensive scores in the AHP in order to increase the reliability of the chromatographic evaluation results. To examine the direct and indirect effects of warming on the score of comprehensive ornamental value, the partial least squares structural equation model (PLS-SEM) was conducted based on the results of principal component analysis using Smart PLS 3.3.9 (SmartPLS GmbH, Monheim am Rhein, NRW, Germany).

## 5. Conclusions

In summary, short-term warming reduced sexual reproduction and the ornamental value of *I. oxyanthera*. It is implied that the population size of this species may be reduced, and they will spread to higher altitudes under future climate warming; therefore, the protection for these wild flowers should be strengthened. The results of this study also suggest that it is not suitable to directly introduce *I. oxyanthera* to places with higher temperatures unless there are cooling measures in these places or it is feasible to gradually domesticate to enhance its adaptability to high temperatures. In this study, the effects of climate warming on plants were clarified from the aspects of plant morphology and floral characteristics, which provided a theoretical basis for the protection of wild flowering plants and a new perspective and idea for biodiversity to climate change. In the future, we should increase experiments to uncover the specific mechanisms behind the observed effects and to investigate genetic or epigenetic factors influencing the plant’s response to warming. *Impatiens* L. has rich species and high ornamental value; thus, a comparative study of multiple species should be conducted due to species-specific responses of these wild flowers to warming. Meanwhile, it is also necessary to screen species that are more adaptable to warming, providing reference for introduction and cultivation.

## Figures and Tables

**Table 1:** Individual flower longevity, flowering duration and flower number per plant of *I. oxyanthera* under the control and warming treatment in 2017 and 2018. W, the effect of warming; Y, the effect of year; W × Y, the interaction effect of warming and year. Different lowercases represent a significant difference among the four experimental treatments. NS, no significance; *, *p* < 0.05; **, *p* < 0.01; ***, *p* < 0.001.

Trait	2017		2018		W	Y	W × Y
	Control	Warming	Control	Warming			
Male phase (d)	2.417 ± 0.072 ab	2.185 ± 0.068 b	2.898 ± 0.093 a	2.583 ± 0.085 ab	NS	*	NS
Female phase (d)	1.065 ± 0.051 a	1.028 ± 0.051 a	1.333 ± 0.068 a	1.213 ± 0.052 a	NS	NS	NS
Flower longevity (d)	3.481 ± 0.083 b	3.213 ± 0.076 b	4.231 ± 0.085 a	3.796 ± 0.070 ab	NS	**	NS
Flowering duration per plant (d)	65.278 ± 1.474 a	58.944 ± 1.330 b	65.917 ± 1.899 a	53.361 ± 1.840 c	**	NS	**
Flower number per plant (No.)	80.778 ± 5.155 a	73.306 ± 4.251 b	73.472 ± 4.357 b	65.500 ± 5.361 c	***	***	NS

**Table 2:** Floral morphology and relative anthocyanin content of *I. oxyanthera* under the control and warming treatment in 2017 and 2018. W, the effect of warming; Y, the effect of year; W × Y, the interaction effect of warming and year. Different lowercases represent a significant difference among the four experimental treatments. NS, no significance; *, *p* < 0.05; **, *p* < 0.01; ***, *p* < 0.001.

Trait	2017		2018		W	Y	W × Y
	Control	Warming	Control	Warming			
Vexillum length (mm)	12.776 ± 0.245 a	11.840 ± 0.213 b	12.546 ± 0.101 a	12.181 ± 0.152 ab	***	NS	NS
Wing petal length (mm)	23.058 ± 0.240 ab	22.289 ± 0.258 b	23.650 ± 0.263 a	22.807 ± 0.217 ab	*	NS	NS
Corolla diameter (mm)	21.916 ± 0.407 b	21.626 ± 0.360 b	23.434 ± 0.361 a	22.465 ± 0.384 ab	NS	**	NS
Corolla tube length (mm)	20.202 ± 0.310 a	19.275 ± 0.220 b	20.444 ± 0.215 a	18.637 ± 0.252 b	***	NS	NS
Stripe number on the labellum (No.)	11.167 ± 0.232 a	10.713 ± 0.193 a	10.667 ± 0.183 a	10.222 ± 0.186 a	NS	NS	NS
Nectar spur length (mm)	30.079 ± 0.399 a	27.447 ± 0.536 b	28.458 ± 0.410 b	27.820 ± 0.314 b	***	**	**
Nectar spur curvature (°)	303.333 ± 12.626 b	300.000 ± 10.992 b	334.352 ± 10.114 a	326.389 ± 9.408 a	NS	*	NS
Pedicel length (mm)	46.285 ± 1.725 a	38.664 ± 1.292 b	40.055 ± 1.107 b	38.960 ± 1.379 b	***	**	*
Relative anthocyanin content of vexillum (A. g^−1^ FW)	5.624 ± 0.136 a	5.867 ± 0.159 a	5.563 ± 0.120 a	5.851 ± 0.114 a	NS	NS	NS
Relative anthocyanin content of corolla tube (A. g^−1^ FW)	3.659 ± 0.078 a	3.722 ± 0.095 a	3.560 ± 0.056 a	3.723 ± 0.112 a	NS	NS	NS

## Data Availability

Original data are available upon request from the corresponding author.

## Supplementary Materials

The following supporting information can be downloaded at: https://www.mdpi.com/article/10.3390/plants13040511/s1, Figure S1: Changes of air daily mean temperature(a, b), soil temperature at 10-cm depth (c, d) and relative air humidity (e, f) during warming in 2017 and 2018; Figure S2: The standard of measurement for flower characteristics; Figure S3: Average daily precipitation in 2017(a) and 2018(b). Daily precipitation data from the meteorological station at the Emei Mountain Jinding (103.2°N, 29.31°E, 3047.1 m a.s.l.) and Leshan (103.45°N, 29.34°E, 424.2 m a.s.l.); Table S1: Average random consistency index of judgement matrix; Table S2: Judgement matrix and consistency check and weight value of the evaluation factors; Table S3: Temperature and humidity changes during warming in 2017 and 2018. W, the effect of warming; Y, the effect of year; W×Y, the interaction effect of warming and year. Different lowercases represent significant difference among four experimental treatments. NS, no significance; *, *p* < 0.05; **, *p* < 0.01; ***, *p* < 0.001.
