# Supplementary material for: Two Growing-Season Warming Partly Promoted Growth but Decreased Reproduction and Ornamental Value of Impatiens oxyanthera
Source: Plants (Basel). 2024 Feb 12;13(4):511. doi: 10.3390/plants13040511 (PMC10892807; doi:10.3390/plants13040511)
Supplement: Supplementary file 1 [file plants-13-00511-s001.zip › Table S1.pdf]

N	1	2	3	4	5	6	7	8	9	10
RI	0.000	0.000	0.580	0.900	1.120	1.240	1.320	1.410	1.450	1.490
