# Supplementary material for: Two Growing-Season Warming Partly Promoted Growth but Decreased Reproduction and Ornamental Value of Impatiens oxyanthera
Source: Plants (Basel). 2024 Feb 12;13(4):511. doi: 10.3390/plants13040511 (PMC10892807; doi:10.3390/plants13040511)
Supplement: Supplementary file 1 [file plants-13-00511-s001.zip › Table S2.pdf]

Constraint hierarchy C	Weight value W	Consistency check	Standard hierarchy P	Weight value W	Consistency check	Weight of layer P contributed to A
$C1$	0.396		$P1$	0.476	λ_{max} = 4.060<br>CI = 0.020<br>CR = 0.022 < 0.100	0.188
			$P2$	0.268		0.106
			$P3$	0.176		0.07
			$P4$	0.08		0.032
$C2$	0.229		$P5$	0.431	λ_{max}=5.140	0.099
$C3$	0.141	λ_{max} = 7.100<br>CI = 0.017<br>CR = 0.013<0.100	$P6$	0.253	CI = 0.035<br>CR = 0.031 < 0.100	0.058
			$P7$	0.182		0.042
			$P8$	0.09		0.021
			$P9$	0.044		0.01
$C4$	0.108		$P10$		λ_{max}=3.009<br>CI = 0.005<br>CR = 0.009 < 0.100	0.181
$C5$	0.057		$P11$			0.096
$C6$	0.042		$P12$			0.07
			$P13$	0.539		0.015
$C7$	0.027		$P14$	0.297		0.008
			$P15$	0.164		0.004
