# Supplementary material for: Two Growing-Season Warming Partly Promoted Growth but Decreased Reproduction and Ornamental Value of Impatiens oxyanthera
Source: Plants (Basel). 2024 Feb 12;13(4):511. doi: 10.3390/plants13040511 (PMC10892807; doi:10.3390/plants13040511)
Supplement: Supplementary file 1 [file plants-13-00511-s001.zip › Table S3.pdf]

| Trait | 2017 | | 2018 | | W | Y | W × Y |
|---|---|---|---|---|---|---|---|
| | Control | Warming | Control | Warming | | | |
| Air daily mean temperature(℃) | 20.966 ± 0.289 a | 22.859 ± 0.316 b | 20.308 ± 0.292 a | 22.684± 0.339 b | *** | NS | NS |
| Relative air humidity(%) | 91.804 ± 0.715 a | 81.184 ± 0.856 b | 88.865± 0.605 a | 81.619± 0.895 b | *** | ** | * |
| Soil temperature at -10 cm depth(℃) | 19.349 ± 0.239 a | 20.765 ± 0.246 b | 19.134 ± 0.247 a | 20.559 ± 0.274 b | *** | NS | NS |
