# Supplementary figures and images for: Two Growing-Season Warming Partly Promoted Growth but Decreased Reproduction and Ornamental Value of Impatiens oxyanthera
Source: Plants (Basel). 2024 Feb 12;13(4):511. doi: 10.3390/plants13040511 (PMC10892807; doi:10.3390/plants13040511)

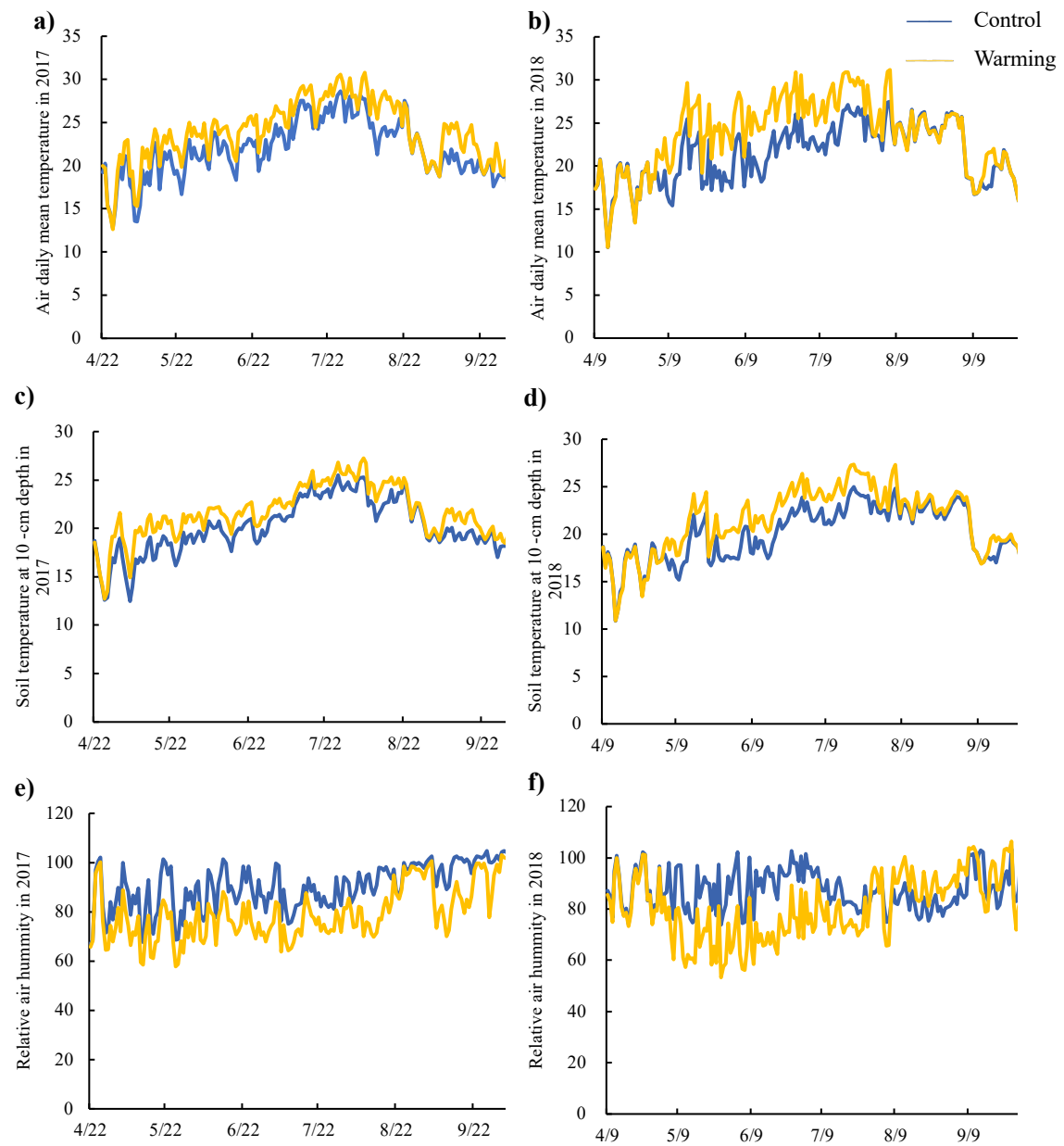

Supplement: Supplementary file 1 [file plants-13-00511-s001.zip › Figure S1.pdf]

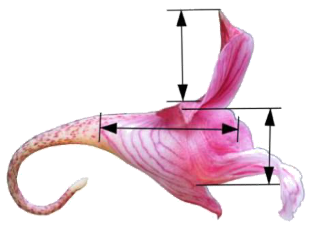

Supplement: Supplementary file 1 [file plants-13-00511-s001.zip › Figure S2(a).png]

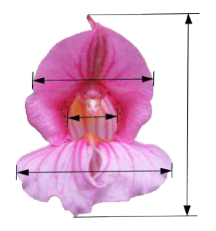

Supplement: Supplementary file 1 [file plants-13-00511-s001.zip › Figure S2(b).png]

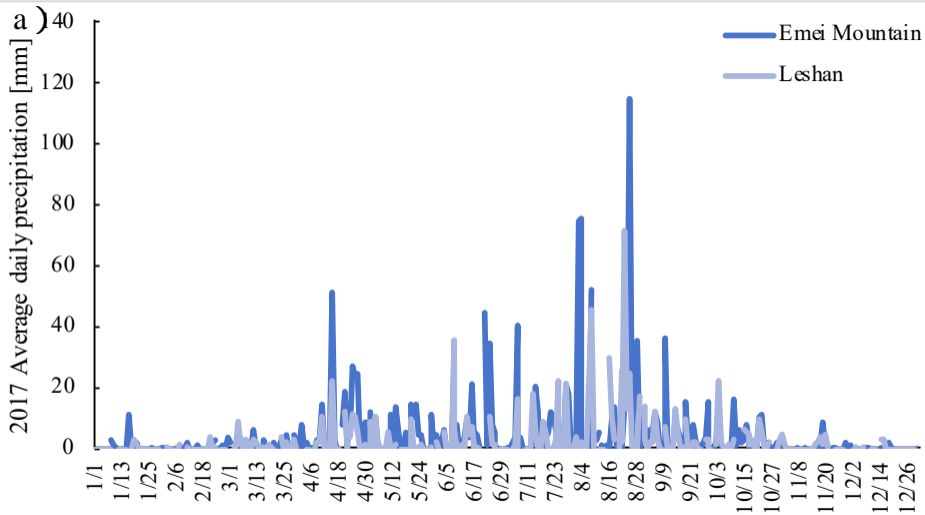

Supplement: Supplementary file 1 [file plants-13-00511-s001.zip › Figure S3(a).pdf]

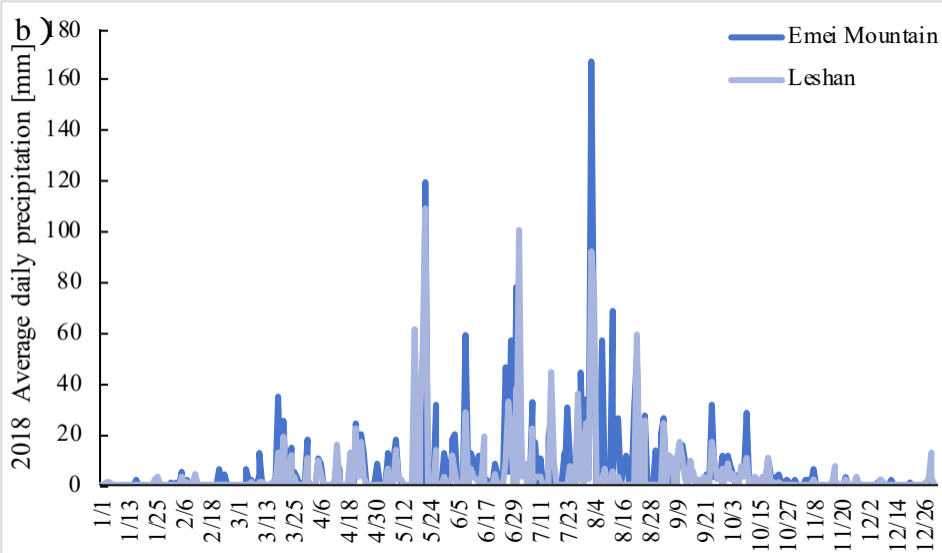

Supplement: Supplementary file 1 [file plants-13-00511-s001.zip › Figure S3(b).pdf]
